# Supplementary material for: Prevalence of congenital missing permanent teeth and its association with side and gender in a Saudi subpopulation
Source: PLoS One. 2025 Sep 25;20(9):e0332958. doi: 10.1371/journal.pone.0332958 (PMC12463198; doi:10.1371/journal.pone.0332958)
Supplement: Attachment — Submitted filename: Reply to Reviewers comments.docx [file pone.0332958.s002.docx]

| **Point- by- Point Reply to Review Comments**  Thank you for your encouraging remarks and for your suggestions, which have improved our manuscript. We were able to address all your suggestions as detailed in the point-by-point reply below: | | |
| --- | --- | --- |
| **Journal Requirements:** | | |
| **Comment** | | **Response** |
| **1** | Please ensure that your manuscript meets PLOS ONE's style requirements, including those for file naming. | Done |
| **2** | Thank you for stating the following financial disclosure:  [This research has been funded by Scientific Research Deanship at University of Ha’il - Saudi Arabia through project number <<RG-23 113>>].  Please state what role the funders took in the study.  If the funders had no role, please state: "The funders had no role in study design, data collection and analysis, decision to publish, or preparation of the manuscript."  If this statement is not correct you must amend it as needed.  Please include this amended Role of Funder statement in your cover letter; we will change the online submission form on your behalf. | This research has been funded by Scientific Research Deanship at University of Ha’il - Saudi Arabia through project number <<RG-23 113>>. The funders had no role in study design, data collection and analysis, decision to publish, or preparation of the manuscript. |
| **3** | When completing the data availability statement of the submission form, you indicated that you will make your data available on acceptance. We strongly recommend all authors decide on a data sharing plan before acceptance, as the process can be lengthy and hold up publication timelines. Please note that, though access restrictions are acceptable now, your entire data will need to be made freely accessible if your manuscript is accepted for publication. This policy applies to all data except where public deposition would breach compliance with the protocol approved by your research ethics board. If you are unable to adhere to our open data policy, please kindly revise your statement to explain your reasoning and we will seek the editor's input on an exemption. Please be assured that, once you have provided your new statement, the assessment of your exemption will not hold up the peer review process. | We agree to make the data available on acceptance. |
| **4** | Please include a copy of Tables 1, 2, and 3 which you refer to in your text on pages 7, 8, 9. | We include the copy of Tables 1 and 2. It appears that Table 3 was mistakenly inserted in place of Table 2. I apologize for the oversight. We correct the error and ensure the appropriate table 2 is included in the revised version. |
| **Reviewer: 1** | | |
| **5** | The written style and results presentation could also have been improved | We appreciate your suggestion regarding the written style and results presentation. We carefully reviewed the manuscript to enhance the clarity and coherence of the writing. Additionally, we revised the presentation of the results to ensure they are more clearly structured and easier to interpret, possibly by refining tables, figures, and descriptive summaries. |
| **6** | One of my main concerns is that the authors observed a prevalence of 13.2%, that is too high once third molar agenesis were not take into consideration. However, this could be explained by the included patients age, which ranged from 9 to 60 years old. To have a correct diagnosis of agenesis in this age is a difficult task. | We acknowledge that the reported prevalence of 13.2% may appear elevated, especially considering that third molar agenesis was not included. As you correctly pointed out, the wide age range of the included patients (9 to 60 years old) may have influenced the results, particularly in the younger subset where dental development is still ongoing. We recognize that diagnosing agenesis accurately in younger patients can be challenging due to variability in tooth development and eruption times. This limitation be more clearly addressed and discussed in the revised version of the manuscript to provide better context for the reported prevalence. |
| **Reviewer: 2** | | |
| 7 | The title is awkwardly phrased. Suggest rewording for clarity:  “Prevalence of Congenital Missing Permanent Teeth and Its Association with Side and Gender in a Saudi Subpopulation”. | Corrected |
| **8** | Abstract: The abstract contains multiple grammatical issues and lacks clarity in the conclusion. Consider stating a more definitive takeaway. The term “most congenital missing” is not standard. Consider using “most commonly missing”. | We have revised the abstract to correct the grammatical issues and improved the clarity of the conclusion by stating a more definitive takeaway. Additionally, the phrase “most congenital missing” has been replaced with the more appropriate term “most commonly missing.” |
| **9** | Methodology: The methodology is sound, but the explanation of examiner calibration and statistical analysis is verbose and could be more concise. It would strengthen the paper to include inter- and intra-examiner reliability. | We have revised the section on examiner calibration and statistical analysis to make it more concise and focused. Additionally, we have included the inter- and intra-examiner reliability results. |
| **10** | Clarify how the classification of agenesis (mild/moderate/severe) was used in the analysis — this is mentioned but not utilized in the results or discussion meaningfully. | Tooth agenesis was categorized based on literature into three groups: mild (one or two missing teeth), moderate (three to five missing teeth), and severe (six or more missing teeth). Additional explanations regarding these classifications were provided in the results section. |
| **11** | Results: Results are described adequately, but tables and figures should be referenced more clearly within the text. There is unnecessary repetition in reporting gender and arch differences in multiple sections. Consider using more precise language in the interpretation of statistical results (e.g., “not statistically significant” rather than “do not statistically significantly correlate”). | Done |
| **12** | Discussion: The discussion is too long and would benefit from being more focused and structured (e.g., prevalence compared to regional and global studies, followed by implications, and then limitations). | Done. We acknowledge that we streamlined the discussion with enhancing clarity and coherence. |
| **13** | Some references are outdated or repeated. Update with more recent literature where available. | We checked the references and added more update one. The repeated references were removed. |
| **14** | The authors should more critically assess potential sources of bias and the retrospective design limitations. | Corrected |
| **15** | Figures & Tables: Ensure figures are high-resolution and labeled appropriately. The figure legends are too vague. Specify which findings each figure is illustrating and provide clearer visual comparisons where applicable. | Done |
| **16** | Replace terms like “congenital missing” with more standard terminology such as “congenitally missing teeth” or “tooth agenesis”. Consider using consistent terminology throughout (e.g., “hypodontia” vs. “agenesis”). | We have revised the manuscript to replace non-standard terms like “congenital missing” with “congenitally missing teeth”.” We have also ensured consistent use of terminology throughout the text, by using “agenesis”. |
